# Anti gC1qR/p32/HABP1 Antibody Therapy Decreases Tumor Growth in an Orthotopic Murine Xenotransplant Model of Triple Negative Breast Cancer

**DOI:** 10.3390/antib9040051

**Published:** 2020-10-06

**Authors:** Ellinor I. Peerschke, Elisa de Stanchina, Qing Chang, Katia Manova-Todorova, Afsar Barlas, Anne G. Savitt, Brian V. Geisbrecht, Berhane Ghebrehiwet

**Affiliations:** 1 Department of Laboratory Medicine, Memorial Sloan Kettering Cancer Center, New York, NY 10065, USA; 2 Sloan Kettering Institute, Memorial Sloan Kettering Cancer Center, New York, NY 10065, USA; destance@mskcc.org (E.d.S.); changq@mskcc.org (Q.C.); manovak@mskcc.org (K.M.-T.); barlasa@mskcc.org (A.B.); 3 Department of Microbiology and Immunology, Renaissance School of Medicine, Stony Brook University, Stony Brook, NY 11794, USA; anne.savitt@stonybrook.edu; 4 Department of Biochemistry and Molecular Biophysics, Kansas State University, Manhattan, KS 66506, USA; geisbrechtb@ksu.edu; 5 Departments of Medicine and Pathology, Renaissance School of Medicine, Stony Brook University, Stony Brook, NY 11794, USA; Berhane.ghebrehiwet@stonybrookmedicine.edu

**Keywords:** gC1qR, breast cancer, xenotransplant model

## Abstract

gC1qR is highly expressed in breast cancer and plays a role in cancer cell proliferation. This study explored therapy with gC1qR monoclonal antibody 60.11, directed against the C1q binding domain of gC1qR, in a murine orthotopic xenotransplant model of triple negative breast cancer. MDA231 breast cancer cells were injected into the mammary fat pad of athymic nu/nu female mice. Mice were segregated into three groups (*n* = 5, each) and treated with the vehicle (group 1) or gC1qR antibody 60.11 (100 mg/kg) twice weekly, starting at day 3 post-implantation (group 2) or when the tumor volume reached 100 mm^3^ (group 3). At study termination (d = 35), the average tumor volume in the control group measured 895 ± 143 mm^3^, compared to 401 ± 48 mm^3^ and 701 ± 100 mm^3^ in groups 2 and 3, respectively (*p* < 0.05). Immunohistochemical staining of excised tumors revealed increased apoptosis (caspase 3 and TUNEL staining) in 60.11-treated mice compared to controls, and decreased angiogenesis (CD31 staining). Slightly decreased white blood cell counts were noted in 60.11-treated mice. Otherwise, no overt toxicities were observed. These data are the first to demonstrate an in vivo anti-tumor effect of 60.11 therapy in a mouse model of triple negative breast cancer.

## 1. Introduction

Triple negative breast cancer is characterized by the absence of estrogen and progesterone receptors, as well as human epidermal growth factor receptor 2 [1,2,3]. Due to the absence of hormone receptors, chemotherapy represents the major therapeutic modality for triple negative breast cancer. The median survival, especially for patients with advanced disease [2,3], remains poor. For this reason, the development of additional therapies directed against novel cellular targets is an important goal to deepen disease response and improve patient outcomes [4,5].

The complement system is emerging as a novel target in cancer therapy. Complement is involved not only in shaping the inflammatory tumor microenvironment, but also in tumor growth and spread [6,7,8,9,10]. In this regard, the complement component C1q is increasingly recognized as a tumor promoting factor, enhancing cancer cell adhesion, migration, proliferation, and angiogenesis [11,12].

We have identified gC1qR (also known as/p32/HABP1) as the major cellular binding site for C1q [13]. Marked upregulation of gC1qR expression has been observed in proliferating cells, particularly in cancers of epithelial cell origin including breast, colon, and lung cancers [14,15]. Moreover, overexpression of gC1qR has been associated with poor prognosis in patients with breast cancer [16,17], prostate cancer [18], serous ovarian adenocarcinoma [19], and endometrial cell cancer [20]. In addition, gC1qR has been identified as a potential molecular target for delivery of cytotoxic agents [21,22].

The present study used a mouse xenograft model to investigate the C1q-gC1qR axis in triple negative breast cancer with the 60.11 murine monoclonal antibody, 60.11, which is directed specifically against the C1q binding domain of gC1qR [23]. Human tumor xenograft models provide important insights into tumor progression and metastasis. We selected the MDA-MB-231 (MDA231) human breast cancer cell line, as it represents a triple negative breast cancer cell line that has been widely studied in xenotransplantation [24]. Moreover, MDA231 cells bind the 60.11 antibody [21], and the role of gC1qR in MDA231 cell proliferation has been described [25,26].

## 2. Materials and Methods

### 2.1. Antibody Production

The therapeutic murine monoclonal antibody (60.11) (IgG) is directed against N-terminal amino acids 76–93 of human gC1qR, and specifically inhibits C1q binding [27,28]. Surface plasmon resonance studies estimate the binding affinity of 60.11 for gC1qR at 67 nM (Appendix A). The antibody recognizes human, mouse, and rat gC1qR [27,28]. Human and rodent (rat/mouse) gC1qR (C1qBP) cDNA sequences are 89.9% identical [29,30].

The study antibody was prepared using in vitro ascites (IVA), as described [31]. Hybridoma 60.11 was cultured in DMEM (Gibco/Thermo Fisher Scientific, Waltham, MA, USA supplemented with 10% Fetal Clone I serum (HyClone, Logan, UT, USA), penicillin and streptomycin (Gibco), and non-essential amino acids (NEAA, Gibco), and subcloned by limiting dilution to identify a high-producing subclone. Hybridoma supernatants were tested by ELISA against recombinant gC1qR antigen. The selected subclone was then adapted into an animal-derived component-free medium (ADCF, HyClone) supplemented with NEAA and inoculated into a CELLine CL1000 flask (Wheaton) according to the manufacturer’s instructions. Antibody-containing supernatants (IVA) were harvested under sterile conditions according to manufacturer’s instructions. Collected supernatants were transferred to sterile tubes (Falcon/Corning Life Sciences, Teterboro, NJ, USA) and stored at −20 °C until used. Antibody quantitation was accomplished by quantitative Western blot. Low-endotoxin, azide-free (LEAF) IgG_1_ kappa (BioLegend, Dedham, MA, USA) was used to generate a standard curve. Antibody was detected in the blot using Alexa Fluor 680-labeled anti mouse IgG (Thermo Fisher, Waltham, MA, USA). Visualization and densitometry were performed on a Licor Odyssey Infrared Imager.

### 2.2. Murine Xenotransplantation Model

An orthotopic xenograft model was used to test the in vivo efficacy of 60.11 antibody therapy, in collaboration with the MSK Antitumor Assessment Core, according to established protocols [32,33,34]. All procedures were performed under approved Institutional Animal Care and Use Committee protocols (04–03–009). Briefly, 5 million MDA231 breast cancer cells (ATCC) were injected into the 4th left mammary fat pad of athymic nu/nu female mice (5–6 weeks old). Animals were treated with gC1qR antibody 60.11 (100mg/kg) starting either 3 days post-MDA231 cell implantation (group 2) before tumors were measurable, or on day 13, after tumor volume reached approximately 100 mm^3^ (groups 1 and 3). Control mice (group 1) were treated with the vehicle, starting 3 days after MDA231 cell implantation. Each treatment group consisted of 5 mice, exposed to twice-weekly intraperitoneal antibody or vehicle injection. Over the course of the experiment, animals in group 2 received 16 doses of 60.11 antibody, whereas animals in group 3 received 11 doses. Animal weights and tumor volumes were recorded twice weekly. Tumor volumes were calculated using the following equation, ((width^2^ × length × 3.14)/6). In addition, clinical assessments of animal distress (weight loss, disruption of locomotor coordination, hunching, lack of grooming, lethargy) were made and recorded daily to assess toxicity. At time of sacrifice (35 days after MDA231 cell implantation), automated blood cell counts (Element HT5 veterinary hematology analyzer, Heska, Loveland, CO, USA) were obtained and tumors were removed, fixed, and processed for histologic (hematoxylin and eosin staining) and immunohistochemical evaluation. In addition, vital organs were harvested for histologic examination. Serum 60.11 antibody levels were quantified using a solid-phase ELISA assay using immobilized recombinant gC1qR and 60.11 antibody standards [35].

### 2.3. Immunohistochemical Analysis

Tissue processing and immunohistochemical analysis was performed by the Molecular Cytology Core Facility of Memorial Sloan Kettering Cancer Center as previously described [36,37]. In brief, tissues were fixed in 4% formaldehyde and processed by paraffin embedding, using a tissue processor (Leica ASP6025). Next, 5 μm sections were obtained and applied to superfrost plus slides. Immunohistochemical detection of Ki 67, Cleaved Caspase 3, TUNEL (Terminal deoxynucleotidyl dUTP nick end labeling), and CD31 was performed using a Discovery XT processor (Ventana Medical Systems, Oro Valley, AZ, USA). Slides were counterstained with hematoxylin and cover-slipped with Permount (Fisher Scientific).

#### 2.3.1. Ki 67 Immunostaining

The Discovery XT autostainer was programmed to incubate slides with primary rabbit polyclonal Ki 67 antibody (Abcam, Cambridge, MA, USA) at 1 μg/mL for 4 h, followed by incubation with secondary antibody (biotinylated goat anti-rabbit IgG; Vector Labs, San Diego, CA, USA) at a concentration of 5.75 μg/mL for 30 min. Blocker D, Streptavidin—HRP, and DAB detection kit (Ventana Medical Systems) were used according to the manufacturer’s instructions.

#### 2.3.2. Cleaved Caspase 3 Immunostaining

A rabbit polyclonal Cleaved Caspase 3 antibody (Cell Signaling) was used at 0.1 μg/mL concentration. Slides were incubated in the Discovery XT autostainer for 3 h. Incubation with secondary antibody (biotinylated goat anti-rabbit IgG; Vector labs) at a concentration of 5.75 μg/mL occurred for 20 min. Blocker D, Streptavidin—HRP, and DAB detection kit (Ventana Medical Systems, Oro Valley, AZ, USA) were used according to the manufacturer’s instructions.

#### 2.3.3. TUNEL Immunostaining

TUNEL analysis was performed as follows. Slides were manually de-paraffinized in xylene, rehydrated in a series of alcohol dilutions (100%, 95%, and 70%) and tap water, and placed into the autostainer. Tissue sections were treated with Proteinase K (20 μg/mL in PBS) for 8 min, incubated with endogenous biotin blocking kit (Roche Diagnostics, Florham Park, NJ, USA) for 12 min, and incubated with labeling mix: TdT (Roche, 1000 U/mL) and biotin-dUTP (Roche, 4.5 nmol/mL) for 2 h. Detection was performed with Streptavidin—HRP and DAB detection kit (Ventana Medical Systems) according to the manufacturer’s instruction.

#### 2.3.4. CD31 Immunostaining

Primary antibody, a rat anti-mouse CD31 antibody (Dianova, Pine Bush, NY, USA), was used at 2 μg/mL. Slides were incubated in the autostainer for 6 h, followed by exposure to biotinylated rabbit anti-rat IgG (Vector Laboratories, Inc., Burlingame, CA, USA, 1:200 dilution) for 60 min. Blocker D, Streptavidin—HRP, and DAB detection kit (Ventana Medical Systems) were used according to the manufacturer’s instructions.

### 2.4. Quantitative Analysis of Target Staining

Quantitative analysis of immunohistochemical staining was performed using a scanning microscope (Panoramic Flash 250, 3DHisttech, Budapest, Hungary) with image processing analytical software. Findings were confirmed by microscopic evaluation.

## 3. Results

**Figure 1:**
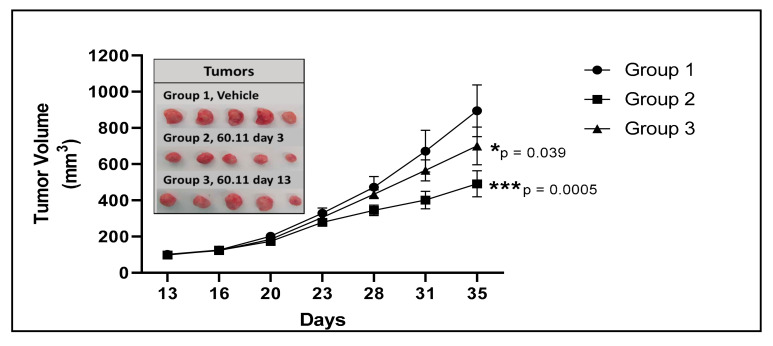
Tumor development in vehicle control and 60.11-treated mice. Figure 1. gC1qR therapy with 60.11 antibody inhibits MDA231 cell proliferation. Tumor volumes of vehicle-treated control mice (group 1) and mice treated with 60.11 antibody are presented over time (35 days). 60.11 therapy was initiated either three days after MDA231 cell implantation (group 2) or on day 13, when tumor volume had reached approximately 100 mm^3^ (group 3). Mean and standard deviation (SD) of tumor volume is shown for each treatment group (*n* = 5 animals per group). *P* values were determined by Student *t*-test. (*) designates statistically significant differences in tumor volume between control and treatment groups (*p* < 0.05). Images of individual tumors resected at study termination are shown in the inset.

Previous studies have shown that gC1qR is upregulated in a variety of breast cancer cell lines including MDA231 triple negative cells [12,25,26], and human breast cancer tumors [14,15]. In the present study, we used the 60.11 monoclonal antibody directed against the C1q binding domain of gC1qR to assess tumor development in mice transplanted with MDA-231 cells. Compared to control mice, animals treated with 60.11 antibody developed smaller tumors (Figure 1, Table 1). A statistically significant difference in tumor volume was noted after 9 doses of 60.11 therapy (day 20) when treatment was initiated 3 days after tumor implantation (group 2), and after 10 doses of 60.11 therapy (day 35) when treatment was initiated after tumors had reached 100 mm^3^ (group 3). Antibody treatment had no effect on mouse weight or physical and behavioral characteristics. Serum 60.11 concentrations, measured at study termination, were variable, with an average of 50 μg/mL (Table 1).

**Figure 2:**
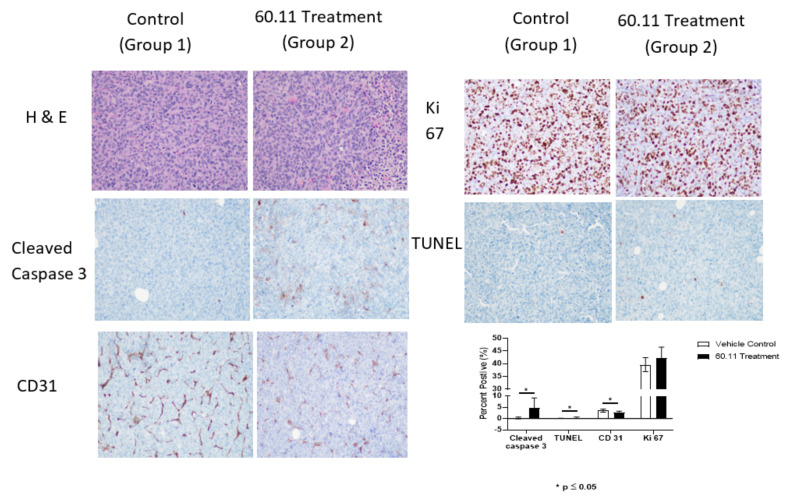
Histologic and immunohistochemical evaluation of MDA231 breast tumors. Figure 2 Representative histologic images (20 × original magnification) of MDA231 tumors obtained from control (group 1) and 60.11-treated (group 2) mice. Sections were stained with hematoxylin and eosin (H&E), cleaved caspase 3, CD31, Ki 76, and TUNEL. Positive immunohistochemical reactivity is represented by brown stain. Quantitative immunohistochemical evaluation is shown in the bar graph. Results represent mean ± S.D. (*n* = 5) of proportional (%) surface area staining positively for the indicated markers. (*) designates statistical significance, *p* < 0.05.

Immunohistochemical studies of excised tumors were performed to gain insight into the mechanism of action of 60.11 therapy. Results were compared between controls and treatment group 2. The data demonstrate an increase in early and late apoptosis markers, cleaved caspase 3, and TUNEL, respectively, in the treatment group (Figure 2). No difference in cell proliferation index (Ki 67) was noted.

Interestingly, 60.11 therapy was associated also with decreased CD31 staining. CD31 is a murine endothelial cell marker that is widely used to assess angiogenesis in tumor models. Since gC1qR has been implicated in angiogenesis [8,25], tumors were stained with CD31 to quantify vascular structures in the developing MDA231 tumors. Fewer CD31-positive structures (brown staining) were observed in tumors from treated mice, and the CD31-positive structures appeared small, as compared to their control counterparts.

Since gC1qR is expressed not only by malignant cells but also by blood cells (B lymphocytes [13], platelets [38], neutrophils [39], eosinophils [40], and macrophages and dendritic cells [41,42]) as well as by proliferating normal cells [15], blood cell counts and vital organs were examined at study termination for on target/off tumor effects. Table 2 compares blood cell counts of control mice and mice with the greatest 60.11 exposure (group 2). Small but statistically significant differences in WBC counts, reflected by decreases in granulocytes (neutrophils, eosinophils, basophils) and lymphocytes, were noted. Reference values are influenced by differences in laboratory instrumentation, methods, collection sites, mouse age, and sex, as well as by environmental factors. Therefore, group comparisons, as shown here (control vs. treatment groups), may be more appropriate than comparisons to reference ranges alone [43].

No evidence of tissue damage was observed by histologic examination of vital organs (Appendix B). In particular, lining cells of the gastrointestinal tract, previously reported to express higher levels of gC1qR than other normal tissue [15], were closely examined and showed no differences between treatment and control groups.

## 4. Discussion

The present study represents the first in vivo proof-of-concept study to evaluate the efficacy of 60.11 monoclonal gC1qR antibody therapy in a murine orthotopic xenotransplant model of triple negative breast cancer. Human gC1qR is a multiligand multicompartmental cellular protein, which is found in the cytosol, plasma membrane, and mitochondria. In addition, soluble forms are released into the surrounding milieu by proliferating cells [21,44,45]. Indeed, in vitro studies by Kandov et al. [12] suggested not only that gC1qR blockade inhibits breast cancer cell proliferation, but also that extracellular, soluble gC1qR enhances cancer cell proliferation via interaction with cell surface-associated C1q. C1q has been detected on breast cancer cells in vitro by flow cytometry [12], and in human tumors by immunohistochemistry [7].

Based on this information, we tested the therapeutic potential of a gC1qR antibody (60.11), which is directed against the C1q binding site of gC1qR (aa 74–282) [23], in an orthotopic xenotransplant mouse model using the MDA231 cell line, which was previously shown to bind the 60.11 antibody [21]. In the absence of formal pharmacokinetic studies, the 60.11 dosing strategy was based on our previous experience in rats [35] and the desire to achieve plasma concentrations in excess of 10 μg/mL, which are required to demonstrate antiproliferative effects in vitro [12]. Serum 60.11 levels at study termination averaged 50 μg/mL, ranging from 28 to 124 μg/mL.

The results demonstrate that 60.11 therapy inhibits MDA231 breast cancer cell proliferation in vivo. When treatment was begun three days after MDA231 cell implantation (group 2), a statistically significant antibody effect was observed after nine doses of antibody therapy (day 20). Differences in tumor volume between controls and treatment group 2 continued to increase for the remainder of the treatment period (15 days). At the time of study termination, day 35, the average tumor size of treated mice in group 2 was 50% smaller compared to controls. Significant reductions in tumor size were also achieved when the 60.11 treatment was begun after visible tumors had formed (group 3). A statistically significant difference in tumor volume was noted after 10 doses of 60.11 therapy (day 35). Treatment with the 60.11 antibody was associated with increased MDA231 tumor cell apoptosis and decreased angiogenesis.

Previous studies have documented that MDA231 breast cancer tumors in mice retain gC1qR expression, and that the intratumoral distribution of gC1qR, when assessed by immunohistochemical staining, is consistent with a cell surface gC1qR expression pattern [46]. Despite the ubiquitous expression of gC1qR by normal cells and tissues, previous studies showed highly selective anti-gC1qR antibody uptake by MDA231 tumors in vivo [46]. These observations are consistent with our finding that 60.11 therapy is not associated with overt toxicities in vital organs.

Animal weights remained constant, and histologic examination of vital organs, showed no pathologic, inflammatory, or degenerative lesions. However, a small decrease in blood leukocyte counts (granulocytes, lymphocytes) was observed in the 60.11 treatment group. Formal toxicity studies are required to further evaluate the on target/off tumor effects of 60.11 antibody therapy. It is important to note that any observations of toxicity may be limited in an immunocompromised mouse model. Therefore, our limited studies explored potential toxicities related to the antiproliferative effect of 60.11 antibody therapy.

gC1qR is involved in a variety of cellular processes [11]. Although this pilot study was performed with a single breast cancer cell line, the results support the concept that gC1qR may play a broader role in breast cancer cell proliferation. The observed 60.11 treatment-induced inhibition of MDA231 cell proliferation via increased apoptosis suggests a direct effect on cell proliferation. These findings are consistent with previous observations demonstrating reduced proliferation of MDA231 breast cancer cells following gC1qR knock-down [26]. Additionally, the present study provides evidence that modulation of MDA231 tumor development by 60.11 treatment may also occur via impaired angiogenesis.

Angiogenesis is essential for tumor growth in vivo. Tumor angiogenesis requires endothelial cell migration into the tumor, followed by endothelial cell organization into hollow tubes that develop into functional blood vessels [47]. C1q is an important factor in endothelial cell tube formation [8]. In the present study, immunohistochemical staining of tumors from 60.11 antibody-treated mice was remarkable, not only for decreased staining with the endothelial cell marker CD31 compared to controls, but also for the presence of much smaller CD31 staining vascular structures.

Results from this pilot study generate several important questions. Pharmacokinetic and pharmacodynamic studies are needed to better understand the therapeutic potential and toxicologic profile of 60.11 therapy. At a mechanistic level, the contribution of antibody inhibition of the cell surface and extracellular gC1qR warrants further exploration. Further, the role of antibody-dependent cytotoxicity in any observed antitumor effect must be considered but cannot be assessed in the present study with immunodeficient mice.

The expanding non-traditional roles of complement have been identified in recent years, including the participation of C1q in cancer [48]. C1q, a constituent of the first component of complement, has been identified in the microenvironment of breast cancer, as well as colon, lung, and pancreatic cancers, in addition to melanoma [7]. Interestingly, C1q localization in the tumor microenvironment appears concentrated on tumor microvascular endothelial cells and stroma, and is independent of peripheral blood C1q levels, suggesting local synthesis. Indeed, the genes for C1q A, B, and C chains are highly expressed in the stroma of human breast cancers, and high expression levels are associated with poor prognosis [49]. Moreover, C1q deficiency has been associated with decreased tumor growth and enhanced survival in a mouse melanoma model [7]. Results from the present study support the concept that blocking C1q–gC1qR interactions may represent a novel treatment approach in breast cancer, and potentially other malignancies associated with increased gC1qR expression.

## 5. Patents

EP and BG hold a licensed patent for the use of gC1qR antibodies in angioedema and cancer.

## Figures and Tables

**Table 1:** 60.11 therapy reduces MDA231 tumor volume.

	Vehicle	60.11 Treatment (Group 2)	60.11 Treatment (Group 3)
Tumor Volume (mm^3^)	894 ± 143	401 ± 48	700 ± 104
		(*p* = 8.34 × 10^−5^)	(*p* = 0.040)
Mouse Weight (g)	24.80 ± 2.16	25.00 ± 2.00	23.60 ± 1.67
		(*p* = 0.883)	(*p* = 0.356)
Serum 60.11 (μg/mL)	undetectable	52 ± 40	49 ± 25
		(median 34; range 31–124)	(median 47; range 28–89)

Results obtained at study termination (day 35) represent mean ± S.D., *n* = 5. Mice in group 2 were treated with 60.11 antibody, 3 days after MDA 231 cell implantation before tumors were measurable, and received a total of 16 doses of antibody by study termination. Mice in group 3 began 60.11 treatment when tumors were measurable (100mm^3^), and received a total of 11 treatment doses by study termination.

**Table 2:** Comparison of peripheral blood cell counts in vehicle control and 60.11-treated mice.

	Treatment Groups		Reference Values *
Cell Count	Vehicle Control (Group 1)	60.11 Treatment (Group2)	
RBC (10^12^/L)	9.77 ± 0.05	9.68 ± 0.41 (*p* = 0.779)	7.4–10.1
Hgb (g/dL)	15.95 ± 0.81	15.58 ± 0.54 (*p* = 0.441)	13.2–18.0
Platelets (10^9^/L)	769 ± 133	778 ± 137 (*p* = 0.923)	659–1372
WBC (10^9^/L)	7.38 ± 3.50	5.09 ± 1.40 (*p* = 0.005)	2.1–11.3
Neutrophils (10^9^/L)	2.10 ± 0.93	1.42 ± 0.44 (*p* = 0.064)	0.4–2.1
Lymphocytes (10^9^/L)	4.98 ± 2.54	3.46 ± 1.07 (*p* = 0.002)	0.7–9.3
Monocytes (10^9^/L)	0.20 ± 0.10	0.106 ± 0.052 (*p* = 0.42)	0.01–0.43
Eosinophils (10^9^/L)	0.090 ± 0.24	0.094 ± 0.017 (*p* = 7 × 10^−5^)	0–0.4
Basophils (10^9^/L)	0.010 ± 0.005	0.006 ± 0.005 (*p* = 2 × 10^−5^)	0–0.03

Results represent mean ± S.D., *n* = 5. * Reference values reflect locally established ranges for athymic nude mice. Blood was obtained from the retro-orbital plexus before sacrifice.

**Figure A1:**
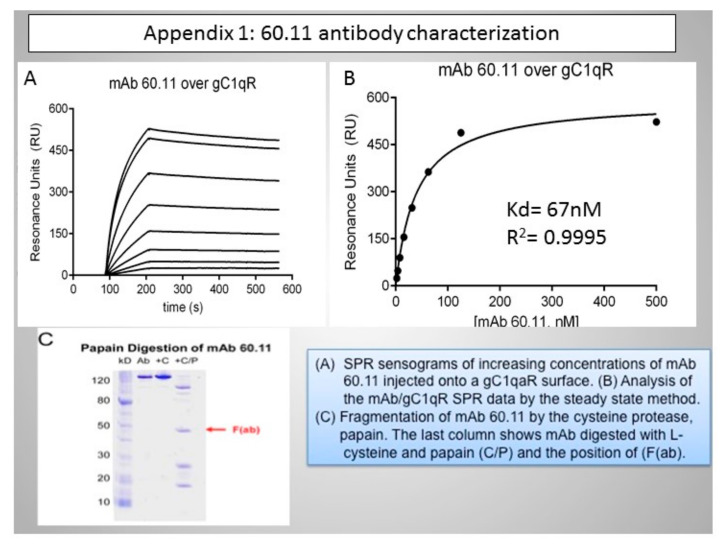
60.11 antibody characterization.

**Figure A2:**
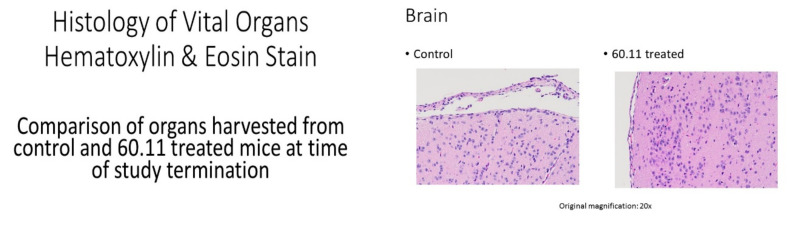

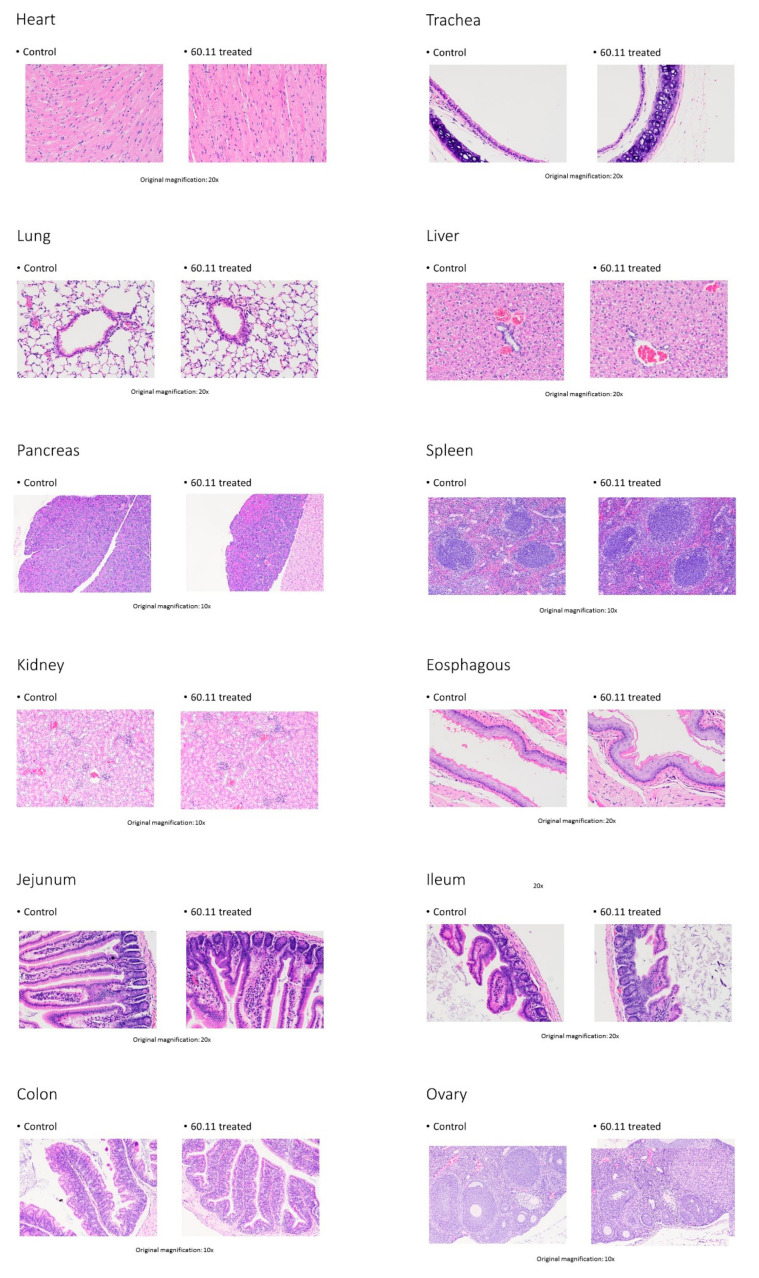
Representative histology of vital organs from control and 60.11 treated animals.
